# Comparison on the Surface Structure Properties along with Fe(II) and Mn(II) Removal Characteristics of Rice Husk Ash, Inactive* Saccharomyces cerevisiae* Powder, and Rice Husk

**DOI:** 10.1155/2016/7183951

**Published:** 2016-11-30

**Authors:** Zhao Jiang, Bo Cao, Guangxia Su, Yan Lu, Jiaying Zhao, Dexin Shan, Xiuyuan Zhang, Ziyi Wang, Ying Zhang

**Affiliations:** School of Resource and Environment, Northeast Agricultural University, Harbin 150030, China

## Abstract

This study selected solid wastes, such as rice husk ash (RHA), inactive* Saccharomyces cerevisiae *powder (ISP), and rice husk (RH), as the potential adsorbents for the removal of Fe(II) and Mn(II) in aqueous solution. The structural characteristics, functional groups, and elemental compositions were determined by scanning electron microscope (SEM) and Fourier translation infrared spectrum (FT-IR) analyses, respectively. Then the influence on the Fe(II) and Mn(II) removing efficiency by the factors, such as pH, adsorbent dosage, initial Fe(II) and Mn(II) concentration, and contact time, was investigated by the static batch test. The adsorption isotherm study results show that Langmuir equation can better fit the Fe(II) and Mn(II) adsorption process by the three adsorbents. The maximum adsorption amounts for Fe(II) were 6.211 mg/g, 4.464 mg/g, and 4.049 mg/g by RHA, ISP, and RH and for Mn(II) were 3.016 mg/g, 2.229 mg/g, and 1.889 mg/g, respectively. The adsorption kinetics results show that the pseudo-second-order kinetic model can better fit the Fe(II) and Mn(II) adsorption process. D-R model and thermodynamic parameters hint that the adsorption processes of Fe(II) and Mn(II) on the three adsorbents took place physically and the processes were feasible, spontaneous, and exothermic.

## 1. Introduction

At present, a lot of effective separation technologies are used to remove heavy metals from aqueous solutions. Among these technologies, adsorption is the most common technique for the removal of heavy metal. It can be used for the removal of pollutants, such as metal ions and organic compounds, from wastewater [1–3]. More interests have arisen in the investigation of adsorbents with a good sorption capacity to remove heavy metal ions from wastewater [4, 5], Therefore, many low-cost agricultural wastes, such as fly ash, natural zeolite, wheat bran, bark and sawdust, peanut shells, rice husk, and cow bone, have been gradually developed for heavy metals removal from aqueous solution [6–9].

The pollution of water resources due to the indiscriminate disposal of heavy metals has been causing worldwide concern over the last few decades. It has been proved that some metals, such as chromium (Cr), copper (Cu), lead (Pb), mercury (Hg), manganese (Mn), cadmium (Cd), nickel (Ni), zinc (Zn), and iron (Fe), could be concentrated in human beings throughout the food chain and affect the health of human beings seriously [10, 11]. Fe and Mn often occur in groundwater in some regions of the world and influence the quality of groundwater with aesthetic, organoleptic, and operating problems [12, 13]. Therefore, the secondary standard maximum contaminant levels (MCLs) of China for iron and manganese in groundwater are 0.3 mg/L and 0.1 mg/L, respectively. It becomes necessary to remove the excess iron and manganese from aqueous solution by an appropriate treatment technology before selecting the water as drinking water or releasing it into the environment [14].

In this study, three solid wastes, such as rice husk ash (RHA), inactive* Saccharomyces cerevisiae* powder (ISP), and rice husk (RH), were selected as the potential adsorbents for the removal of Fe(II) and Mn(II) from aqueous solution. The structure and chemical groups on the surface of the adsorbents mentioned above were investigated by scanning electron microscope (SEM) and Fourier translation infrared spectroscopy (FT-IR) analysis. Meanwhile, the effects of some important sorption parameters, such as pH, contact time, initial metal concentration, and adsorbent dosage, on the removal of Fe(II) and Mn(II) were examined. In addition, the equilibrium data were analyzed using Langmuir, Freundlich, and D-R isotherm models and the dynamic adsorption data was analyzed by pseudo-first-order and pseudo-second-order equations. Finally, the adsorption mechanisms of Fe(II) and Mn(II) ions onto three studied adsorbents were also evaluated in terms of thermodynamics. All these results could help us to evaluate the potential and relative mechanism of the three investigated agricultural wastes in removing the heave metal from aqueous solution.

## 2. Materials and Methods

### 2.1. Adsorbents

Three different biomaterials, such as RHA, ISP, and RH, were collected from rice mill, brewery, and agricultural farm, respectively, which were located in Harbin City, China. The collected biomaterials were sieved through a 0.25 mm mesh and washed several times with distilled water subsequently. The biomaterials were oven dried at 60°C until constant weights were attained and served as the adsorbents for further research.

### 2.2. The Structural Characteristics and Elemental Compositions of the Adsorbents

(1) Surface structure analysis: an ion sputtering apparatus (E-1010, HITACHI) was used to plate 1500 nm thickness of gold film on the surface of the researched adsorbents. The prepared samples were observed by SEM (S-3400N, HITACHI). (2) Chemical group detection: the spectra of the adsorbents prepared as KBr discs were recorded at the wave number of 400–4000 cm^−1^ obtained from a FT-IR spectrometer (Spectrum 2000, Perkin Elmer).

### 2.3. Batch Adsorption Procedure

Batch adsorption studies were carried out by shaking 150 mL flasks containing 50 mL of desired aqueous solution with Fe(II) or Mn(II) at 130 rpm and 25°C. The mixture was filtered using an acid-cleaned 0.45 *μ*m filter and the concentration of Fe(II) and Mn(II) in the filtrate was determined by atomic absorption spectrometry according to the method reported by Unsal et al., [15]. The effects of some important parameters, such as pH, contact time, adsorbent dosage, and initial concentration, on the Fe(II) and Mn(II) removal by the three test adsorbents were researched as follows: (1) the initial pH of the solution containing metal ions was ranging from 1.0 to 8.0 by adjusting with 0.1 mol/L HCl or NaOH; (2) the effect of contact time on the removal of Fe(II) and Mn(II) was studied by taking out the samples from the shaker every 10 min till equilibrium was reached; (3) adsorbent doses ranging from 0.2 g/50 mL to 1.5 g/50 mL were selected to investigate the metal ions removal effect by different doses of adsorbents; (4) the various initial metal concentrations were designed from 5 mg/L to 40 mg/L. Other absorption processes and experimental conditions were similar with those mentioned above. All experiments were repeated three times, and results presented are consequently the averaged values of replicate tests.

### 2.4. Adsorption Isotherm and Kinetic Model

Adsorption isotherms were conducted at adsorbent dose of 0.5 g/50 mL solutions where Fe(II) and Mn(II) concentration was ranging from 2 mg/L to 40 mg/L. The pH of the solutions described above was adjusted to 5.0 and 6.0, respectively. The mixtures were shaken at 130 rpm for 3 hours (25°C). In addition, the kinetic experiments were performed in continuously stirred flask containing 50 mL of 20 mg/L Fe(II) and Mn(II) solutions (pH values were 5 and 6, resp.) and 1 g adsorbent. The mixtures were shaken at 130 rpm (25°C). 5 mL of the mixtures was extracted from 5 min to 90 min. The Fe(II) and Mn(II) concentrations of the mixtures both in isotherm and kinetics were determined after filtering by the methods mentioned in Section 2.3.

The removal percentage (*R*) and equilibrium adsorption amount (*q*_*e*_) of Fe(II) and Mn(II), *q*_*e*_ (mg/g), were calculated by (1) and (2), respectively.

(1) R=100C0−CeC0,

(2) qe=C0−CeVW,

where *C*_0_ is the initial concentration of metal ion in solution (mg/L) and *C*_*e*_ is the equilibrium concentration of Fe(II) or Mn(II) (mg/L).* V* is the volume of the solution (L) and* W* is the mass of the adsorbent (g).

Langmuir, Freundlich, and D–R isotherm models were selected to fit the isotherm parameters of the three adsorbents. Langmuir model is described as (3) and (4) and Freundlich model is described as (4). Furthermore, D–R model is described as (6), and the sorption free energy (*E*) was evaluated by (7).

(3) $$\begin{array}{c} \frac{C_{e}}{q_{e}}=\frac{1}{q_{m}k_{a}}+\frac{C_{e}}{q_{m}}, \end{array}$$

where *C*_*e*_ and *q*_*e*_ are the equilibrium concentration of metal ion in solutions (mg/L) and adsorbent (mg/g), respectively, and *q*_*m*_ is the adsorption capacity of the adsorbent (mg/g).

(4) $$\begin{array}{c} \log q_{e}=\log K_{F}+\frac{1}{n}\log C_{e}, \end{array}$$

where *K*_*F*_ is the Freundlich constant of the relative adsorption capacity of the adsorbent.

(5) $$\begin{array}{c} \ln q_{e}=\ln q_{m}-\beta \varepsilon^{2}, \end{array}$$

where *C*_*e*_ and *q*_*e*_ are the equilibrium concentration of Fe(II) and Mn(II) in solutions (mg/L) and adsorbent dose (mg/g), respectively. *q*_*m*_ is the adsorption capacity of the adsorbent (mg/g). *ε* in (5) could be calculated by (6) as follows:

(6) ε=RTIn⁡1+1Ce,

(7) E=1−2β.

In this work, pseudo-first-order kinetic model and pseudo-second-order kinetic model were employed to analyze the kinetic adsorption and relative parameters. The two models were described as (8) and (9).

(8) $$\begin{array}{c} \ln\left(\;q_{e}-q_{t}\;\right)=\ln q_{e}-k_{1}t, \end{array}$$

where *q*_*e*_ and *q*_*t*_ are the adsorptive capacity (mg/g) at equilibrium and the various sample time *t* (min), respectively, and *k*_1_ is the pseudo-first-order adsorption rate constant (min^−1^).

(9) $$\begin{array}{c} \frac{t}{q_{t}}=\frac{1}{K_{2}{q_{e}}^{2}}+\frac{t}{q_{e}}, \end{array}$$

where *k*_2_ (g/mg min) is the adsorption rate constant of the second-order equation, *q*_*t*_ (mg/g) is the adsorption amount at the sample time *t* (min), and *q*_*e*_ is the adsorption amount of adsorption equilibrium (mg/g).

### 2.5. Adsorption Thermodynamics

The thermodynamic parameters, such as Gibbs free energy (Δ*G* ^0^), enthalpy (Δ*H* ^0^), and entropy (Δ*S* ^0^), were used to describe the thermodynamic behavior of Fe(II) and Mn(II) onto three adsorbents. The parameters mentioned above were calculated by (10).

(10) ΔG0=−RTIn⁡ KD,ln⁡KD=ΔS0R−ΔH0RT,

where *R* is the universal gas constant (8.314 J/mol K), *T* is the temperature (*K*), and *K*_*D*_ is the distribution coefficient which can be calculated by *K*_*D*_ = *q*_*e*_/*C*_*e*_ [16]. *q*_*e*_ and *C*_*e*_ have been described in (3).

## 3. Results and Discussion

### 3.1. Surface Structure and Functional Group Detection

**Figure 1:**
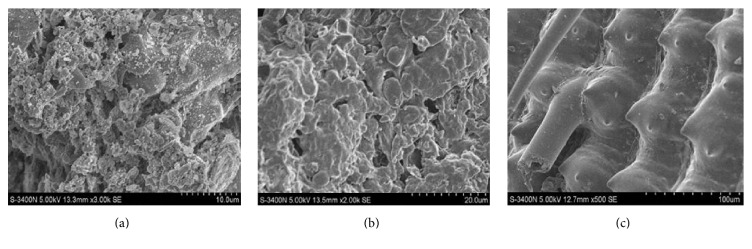
SEM image of the researched adsorbents. (a) Rice husk ash, RHA; (b) inactive* Saccharomyces cerevisiae* powder, ISP; rice husk, RH.

The surface structures of the three potential adsorbents were showed in Figure 1. Obviously the surface of RHA is porous and with numerous honeycomb holes (Figure 1(a)). In addition, the internal structure of RHA possesses a number of irregular pieces of layered and reticulate structure. ISP used in this research is the by-product of beer production and composed of some kinds of organics, such as carbohydrates and protein. As a result, ISP showed different surface structure properties with those of RHA in Figure 1(b). Specifically, the surface of ISP is without clear and concrete structure and exhibits uneven surface state that contains some holes occasionally.  Figure 1(c) showed that there were some regular framework structures on the surface of the RH, and there were many embossments similar to cone between the two lines of framework.

The key chemical groups or bonds of the adsorbents detected by Fourier translation infrared spectrogram were listed in Table 1. It was obvious to show that hydroxyl group, C-H bond, was the main chemical group of RHA. Meanwhile, hydroxyl group, methyl group, and methylene group were detected on the surface of ISP, as well as C-N bond, carbonyl group, N-H bond, and C-N bond. Furthermore, hydroxyl group, ester group, and methyl group were detected in RH besides carbonyl group stretching from aldehydes and ketones based on the peaks located at 1638 cm^−1^[17].

It has been well known that the surface structural properties and the functional groups composition are the two key factors that determine whether the material could serve as a kind of adsorbents. Though the surface structural properties of the studied three-potential adsorbents were different from each other, it is similar that any of the three adsorbents has porous structure (Figure 1). These special surface structural properties could help these adsorbents increase their surface area and exhibit good adsorption capacity. In most cases, the metal ion carried positive charge; therefore, the metal ion was ready to combine with the functional groups mentioned above according to complex electrostatic adsorption process. Overall, the surface characteristics mentioned above hint that the researched biomaterials were suitable to serve as adsorbent to remove the metal ions from solution.

### 3.2. Static Batch Test

#### 3.2.1. Initial pH

**Figure 2:**
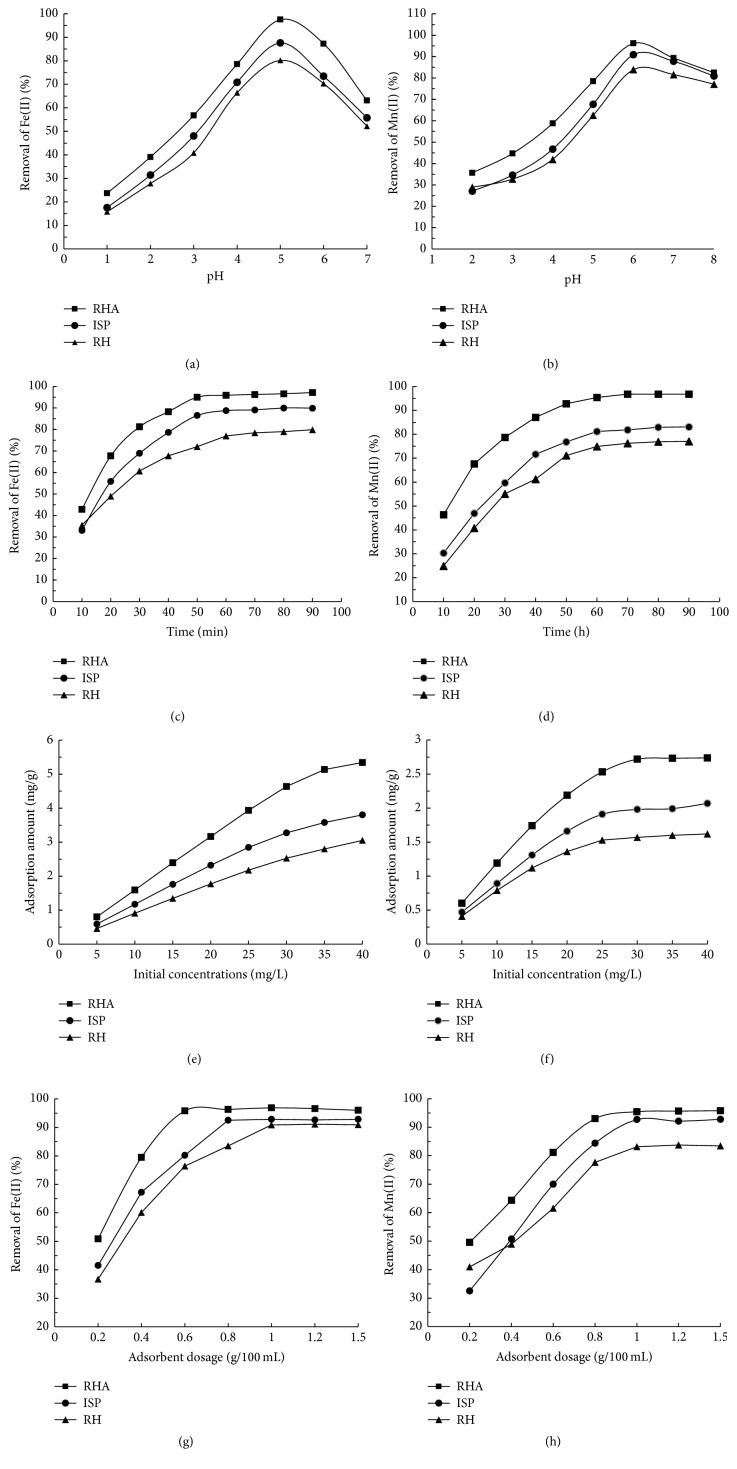
The Fe(II) and Mn(II) adsorption characteristics of the three adsorbents at different (a-b) pH ranged from 1 to 8; (c-d) contact time at 5 min, 10 min, 20 min, 30 min, 40 min, 50 min, 60 min, 70 min, 80 min, and 90 min; (e-f) initial metal ion concentrations are 5 mg/L, 10 mg/L, 15 mg/L, 20 mg/L, 25 mg/L, 30 mg/L, 35 mg/L, and 40 mg/L; (g-h) adsorbents dosage at the levels of 0.2 g/100 mL, 0.4 g/100 mL, 0.6 g/100 mL, 0.8 g/100 mL, 1.0 g/100 mL, 1.2 g/100 mL, and 1.5 g/100 mL. In addition, (a), (c), (e), and (g) indicate the results about the Fe(II) removal and (b), (d), (f), and (h) show the removal of Mn(II). All the results mentioned above were given as the mean of the triplicate.

It has been reported that the initial pH of the solution could affect the adsorption of metal ions on adsorbents [18]. In this paper, the effects of pH on the adsorption of Fe(II) and Mn(II) onto three adsorbents were studied and the results were presented in Figures 2(a) and 2(b). It was observed that the removal amount of Fe(II) and Mn(II) was increased obviously when the initial pH of the metal solution was increasing from 1 to 4 and from 2 to 5, respectively. The maximum removal amounts for Fe(II) were found to be 2.44 mg/g, 2.19 mg/g, and 2.01 mg/g onto RHA, ISP, and RH when initial pH was 5 and the maximum removal amounts for Mn(II) were 1.924 mg/g, 1.818 mg/g, and 1.678 mg/g at pH 6, respectively.

It is well known that the amount of hydrogen ion (H^+^) decides the pH of the solution and that the amount of H^+^ is different among the solutions with various pH values. As a result, the different metal ion removal ability of the adsorbents mentioned above in the solution with various initial pH values might partly be because the two kinds of ions with positive electrical charge, such as metal ions and H^+^, were in contention to contact with the limited active sites on the adsorbent surface [19, 20]. Specifically, because the amount of H^+^ was decreasing when the pH was increasing, much more active sites on the adsorbent surface could be used to adsorb the Fe^2+^ or Mn^2+^. That might be the reason for why the Fe^2+^ and Mn^2+^ removal amount was increased when the initial pH solution was increasing. However, the adsorption efficiency decreased after attaining the maximum adsorption capacity of the adsorbents. This could be due to the formation of soluble hydroxylated complexes of the metal ions and their ionized nature [21]. In order to avoid the precipitation of Fe^2+^ and Mn^2+^, further researches about such two ions adsorption on the three potential adsorbents were carried out with pH values of 5 and 6, respectively.

#### 3.2.2. Contact Time

The reaction time is one of the important factors that influence the adsorption process of heavy metals by adsorbent [22]. Figures 2(c) and 2(d) showed the effect of contact time on the adsorption of Fe(II) and Mn(II) onto three potential adsorbents. The removal percentage of Fe(II) and Mn(II) was increasing gradually when the contact time was prolonged from 5 min to 60 min. On the contrary, there was no considerable increase in Fe(II) and Mn(II) adsorption when the contact time continually prolonged to 80 min. During this period, the highest adsorption percentages for Fe(II) were 97.2%, 89.9%, and 79.9% onto RHA, ISP, and RH, respectively; meanwhile, there were about 96.8%, 83.1%, and 77.0% of Mn(II) removed by the adsorbents described above, respectively. It was found that the adsorption volume of RHA for Fe(II) or Mn(II) was obviously higher than those of ISP and RH. It is reasonable to infer that the different adsorption volume of those three adsorbents might be due to the different composition or adsorption sites of the adsorbents. This inference was supported by the experimental results that there were no obvious differences in Fe(II) or Mn(II) adsorption during the initial stage of sorption period. It is because there were a large number of vacant surface sites on the adsorbents for the metal ions. However, the Fe(II) and Mn(II) removal abilities of the adsorbents were different when approaching adsorption equilibrium which indicates the saturation of the active sites. It also hints that the active adsorption site on the adsorbents was limited [23].

#### 3.2.3. Initial Fe(II) and Mn(II) Concentration

The effects of initial Fe(II) and Mn(II) concentration on the removal of these kinds of metal ion were represented in Figures 2(e) and 2(f). The results suggest that there is much more Fe(II) and Mn(II) that could be removed by the adsorbents when the initial ion concentration was increased from 5 mg/L to 40 mg/L. Specifically, the adsorption amount of Fe(II) by RHA, ISP, and RH was increased from 0.80 mg/g, 0.59 mg/g, and 0.46 mg/g to 5.34 mg/g, 3.80 mg/g, and 3.05 mg/g, respectively; meanwhile, the Mn(II) adsorption amount of the adsorbents described above was increased from 0.23 mg/g, 0.18 mg/g, and 0.16 mg/g to 2.74 mg/g, 2.07 mg/g, and 1.62 mg/g, respectively. Therefore, RHA had the greatest Fe(II) and Mn(II) adsorption capacity among the three adsorbents.

#### 3.2.4. Adsorbent Dose

Figures 2(g) and 2(h) showed that the removal percentage of both metal ions by the three adsorbents was increased exponentially when the dosage of the adsorbent was increased from 0.6 g/100 mL to 1.0 g/100 mL. This phenomenon could further support the previous inference in Section 3.2.2 that there was limited active adsorption site on the three adsorbents studied in this research. In other words, the increasing dosage of adsorbents gives much more active adsorption site to Fe(II) and Mn(II) and enhances the removal percentage of these two kinds of metal ions accordingly. However, these three adsorbents exhibited various Fe(II) and Mn(II) removal abilities; the Fe(II) removal percentages were 96.3%, 92.5%, and 90.9% by RHA, ISP, and RH at the added dosage of 0.6 g/100 mL, respectively; meanwhile, there were about 95.81%, 92.78%, and 83.75% of Mn(II) removed by these adsorbents, respectively, when the adsorbent dosage was 0.8 g/100 mL. The Fe(II) and Mn(II) removal percentage of the three adsorbents changed slightly when their added dosage increased continually. Similarly, Uluozlu et al. reported that the maximum biosorption for Pb(II) and Cr(III) was attained when the adsorbent dosage was 0.4 g/100 mL and that the metal removing amount changed slightly when the adsorbent dosage was increasing from 1 g/100 mL to 2 g/100 mL [21]. These results hint that there might exist an optimal amount of adsorbent for a given initial concentration of heavy metal.

### 3.3. Adsorption Isotherm Models

Adsorption isotherms are used to describe the adsorption process and also could indicate the adsorption behavior and mechanism [24]. The results in Table 2 showed that both Langmuir model and Freundlich model could be fitting well the adsorption process of Fe(II) by the three adsorbents as the *R* ^2^ values were ranging from 0.900 to 0.977 (Table 2). Additionally, the saturated adsorption amounts of Fe(II) were 6.211 mg/g, 4.464 mg/g, and 4.049 mg/g by RHA, ISP, and RH, respectively. It also could be concluded that the Fe(II) adsorption ability of RHA was higher than the other two adsorbents based on *K*_*F*_ and 1/*n* in Freundlich equation (Table 2); two parameters expressed adsorption capacity and adsorption intensity, respectively. Similarly, the higher equilibrium constant Ka in Langmuir equation also presented the higher Fe(II) adsorption capacity of RHA. In addition, Langmuir model also well fitted the Mn(II) adsorption process by RHA, ISP, and RH that the correlation coefficients were 0.986, 0.994, and 0.990 (Table 2). The maximum adsorption capacity of these three adsorbents for Mn(II) was 3.016 mg/g, 2.229 mg/g, and 1.899 mg/g, respectively (Table 2). All these results mentioned above suggest that RHA exhibits excellent adsorption capacity for Fe(II) and Mn(II) compared to that of ISP and RH. Meanwhile, the Fe(II) and Mn(II) adsorption capacity of the three adsorbents, especially for RHA, was also better than those of other previous reported adsorbents, such as fly ash produced by* Aspergillus niger* and manganese oxide coated zeolite with the adsorption capacity for Fe(II) and Mn(II) being 2.01 mg/g and 1.1 mg/g, respectively, [25, 26]. In addition, it has been reported that Langmuir model could be applied to assume the adsorption of metal ions is a kind of monolayer sorption on the surface of adsorbent [27]. Therefore, it is reasonable to infer that the adsorption for Fe(II) and Mn(II) by these adsorbents might be a surface-adsorption process.

D–R isotherm model could be used to predict or determine the nature of adsorption processes as physical or chemical [28]. It also has been proved that the adsorption could be determined as a chemical processes when its adsorption energy (*E*) is between 8 kJ/mol and 16 kJ/mol; otherwise, the adsorption could be considered as a physical process if the *E* value mentioned above was below 8 kJ/mol [29]. The results in Table 2 showed that the adsorption energy (*E*) was calculated as 2.59 kJ/mol, 0.91 kJ/mol, and 0.43 kJ/mol for the adsorption of Fe(II) and 2.65 kJ/mol, 0.52 kJ/mol, and 0.36 kJ/mol for Mn(II) by RHA, ISP, and RH, respectively (Table 2). Therefore, it is reasonable to conclude that the adsorption processes of Fe(II) and Mn(II) onto the three adsorbents were physical processes in nature.

### 3.4. Adsorption Kinetics

Adsorption kinetic has been defined as one of the important characteristics which could show the adsorption efficiency of the adsorbent [30]. The data in Table 3 suggests that the adsorption of Fe(II) by the RHA and ISP was better fitting to pseudo-second-order model compared with pseudo-first-order kinetic model (*R* ^2^ = 0.995 and 0.992, resp.) and that both of the two models mentioned above well fit the adsorption of Fe(II) by RH and the correlation coefficients (*R* ^2^) were the same as 0.993 (Table 2). In addition, the initial adsorption velocity of RHA to Fe(II) was faster than that of other two adsorbents; meanwhile, RH exhibit lowest adsorption velocity to Fe(II) among the three adsorbents. For Mn(II) adsorption, pseudo-second-order model could be fitting well the adsorption by the three adsorbents (Table 3). Based on all the kinetic parameters mentioned above, pseudo-second-order models were fitting well and the adsorption velocity of Fe(II) and Mn(II) by RHA was the fastest.

### 3.5. Adsorption Thermodynamics

The thermodynamic parameters which were used to describe the thermodynamic behavior of Fe(II) and Mn(II) onto three adsorbents were shown in Table 4. The negative values of Δ*G* ^0^ indicate that the thermodynamic nature of the Fe(II) and Mn(II) adsorption by the researched adsorbents is feasible and spontaneous. In addition, the values of Δ*H* ^0^ were found to be −40.94 J/mol, −95.49 J/mol, and −88.80 J/mol for Fe(II) adsorption, as well as −12.92 J/mol, −83.73 J/mol, and −40.74 J/mol for Mn(II) adsorption by RHA, ISP, and RH, respectively (Table 3). These data suggest that the adsorptions of Fe(II) and Mn(II) by the three adsorbents are an exothermic process. Furthermore, the Δ*S* ^0^ of the Fe(II) and Mn(II) adsorption by the researched adsorbents showed in Table 4 predict that the adsorption mentioned above is an irreversible process.

## 4. Conclusions

In this study, batch adsorption experiments for the removal of Fe(II) and Mn(II) from aqueous solutions have been carried out using three adsorbents, such as RHA, ISP, and RH. The pH of the solution, contact time, adsorbent dosage, and initial concentration were the main factors that could affect the adsorption efficiency of Fe(II) and Mn(II) by the three adsorbents. Langmuir equation fitted well Fe(II) and Mn(II) adsorption process and RHA exhibits the most prominent Fe(II) and Mn(II) adsorption capacity among the researched adsorbents. Similarly, the kinetic research results suggest that the adsorption of Fe(II) and Mn(II) can be well described by the pseudo-second-order model; meanwhile, RHA also exhibits fastest adsorption velocity. The thermodynamic parameters proved the feasible, spontaneous, and exothermic nature of the Fe(II) and Mn(II) adsorption onto three adsorbents. Furthermore, the adsorption process mentioned above was physical sorption process.

## Figures and Tables

**Table 1:** The key chemical groups or bonds detected on the three researched potential adsorbents, such as rice husk ash, RHA; inactive *Saccharomyces cerevisiae* powder, ISP; rice husk, RH.

Wavelength	Chemical groups or bonds	Potential adsorbents		
		RHA	ISP	RH
3500–3200 cm^−1^	Hydroxyl group	+	+	+
795 cm^−1^	C-H bond	+	−	−
2928–2925 cm^−1^	Methyl group	−	+	+
2119 cm^−1^	Methylene group	−	+	−
1453 cm^−1^	C-N bond	−	+	−
1657–1638 cm^−1^	Carbonyl group	−	+	+
1533 cm^−1^	N-H bond	−	+	−
1512–1455 cm^−1^	Ester group	−	−	+

Note: “+” represents the corresponding chemical group detected and “−” represents the chemical group not detected.

**Table 2:** Isotherm model constants and nonlinear regression parameters for fit of Fe(II) and Mn(II) adsorbed on the three kinds of adsorbents.

Metal ions	Isotherm models	Parameters or constants	Adsorbents		
			RHA	ISP	RH
Fe(II)	Freundlich	*K*_*F*_	2.649	1.549	0.948
		1/*n*	0.503	0.510	0.598
		*R* ^2^	0.901	0.900	0.958
	Langmuir	*K*_*a*_ (L/mg)	1.032	0.673	0.332
		*q*_*m*_ (mg/g)	6.211	4.464	4.049
		*R* ^2^	0.995	0.995	0.997
	D-R	*E* (kJ/mol)	2.590	0.910	0.430
		*q*_*m*_	1.360	1.060	0.800
		*R* ^2^	0.502	0.654	0.730

Mn(II)	Freundlich	*K*_*F*_	1.379	0.873	0.527
		1/*n*	0.345	0.379	0.437
		*R* ^2^	0.892	0.918	0.909
	Langmuir	*K*_*a*_ (L/mg)	0.907	0.687	0.320
		*q*_*m*_ (mg/g)	3.016	2.229	1.899
		*R* ^2^	0.986	0.994	0.990
	D-R	*E* (kJ/mol)	2.650	0.520	0.360
		*q*_*m*_	1.380	0.850	0.680
		*R* ^2^	0.480	0.709	0.735

*Note*. The three kinds of adsorbents include the following: rice husk ash, RHA; inactive *Saccharomyces cerevisiae *powder, ISP; rice husk, RH.

**Table 3:** Parameters and kinetic models for Fe(II) and Mn(II) adsorption on three researched adsorbents.

Metal ions	Kinetic models	Parameters	Adsorbents		
			RHA	ISP	RH
Fe(II)	Pseudo-first-order	*q*_*e*,exp_ (mg/g)	1.9430	1.7998	1.6000
		*k*_1_ (min^−1^)	0.0714	0.0691	0.0553
		*q*_*e*,calc_ (mg/g)	2.2182	2.4889	1.7660
		*R* ^2^	0.9810	0.9710	0.9930
	Pseudo-second-order	*k*_2_ (g/mg min)	0.0361	0.0248	0.0260
		*q*_*e*,calc_ (mg/g)	2.2573	2.2321	1.9920
		*h*_0_	0.1841	0.1234	0.1033
		*R* ^2^	0.9950	0.9920	0.9930

Mn(II)	Pseudo-first-order	*q*_*e*,exp_ (mg/g)	1.9360	1.6620	1.5400
		*k*_1_ (min^−1^)	0.0691	0.0760	0.0829
		*q*_*e*,calc_ (mg/g)	2.4717	3.6308	4.7424
		*R* ^2^	0.9740	0.9510	0.9240
	Pseudo-second-order	*k*_2_ (g/mg^.^min)	0.0344	0.0192	0.0156
		*q*_*e*,calc_ (mg/g)	2.2620	2.1790	2.1410
		*h*_0_	0.1760	0.0910	0.0720
		*R* ^2^	0.9970	0.9900	0.9850

*Note*. The three kinds of adsorbents include the following: rice husk ash, RHA; inactive *Saccharomyces cerevisiae *powder, ISP; rice husk, RH.

**Table 4:** Thermodynamic parameters for Fe(II) and Mn(II) adsorption on three adsorbents.

Metal ion	Adsorbent	Δ*G* ^0^ (kJ/mol)			Δ*H* ^0^ (J/mol)	Δ*S* ^0^ (J/mol K)
		303 K	313 K	323 K		
Fe(II)	RHA	−2.81	−2.63	−2.30	−40.94	−15.49
	ISP	−2.34	−1.43	−0.69	−95.49	−31.46
	RH	−3.77	−2.97	−2.42	−88.80	−30.99
Mn(II)	RHA	−2.89	−2.50	−2.30	−12.92	−6.60
	ISP	−2.43	−1.24	−0.78	−83.73	−27.69
	RH	−2.52	−2.26	−1.69	−40.74	−14.91

*Note*. The three adsorbents include the following: rice husk ash, RHA; inactive *Saccharomyces cerevisiae *powder, ISP; rice husk, RH.
